# Corrigendum

**DOI:** 10.1002/ehf2.13408

**Published:** 2021-05-03

In the paper by Yu *et al* ^1^_,_ the contents of the supporting information file were incorrect, and the correct data has now been updated on Wiley Online Library.
